# Modified laparoscopic Sugarbaker repair of a recurrent ileostomy prolapse

**DOI:** 10.1093/jscr/rjac013

**Published:** 2022-02-06

**Authors:** Adele S Orovec, Samuel F Minor, C Marius Hoogerboord

**Affiliations:** 1 Faculty of Medicine, Dalhousie University, Halifax, Nova Scotia, Canada; 2 Department of Surgery, Dalhousie University, Halifax, Nova Scotia, Canada

## Abstract

A 57-year-old male who presented with a recurrent ileostomy prolapse was successfully treated with the modified laparoscopic Sugarbaker procedure. This case demonstrates a novel application of the modified laparoscopic Sugarbaker procedure and provides an alternative option for the surgeon managing this challenging problem of recurrent stomal prolapse.

## INTRODUCTION

Stomal prolapse, defined as full-thickness telescoping of bowel though an intestinal stoma, occurs at a rate of 1–16% and can be intermittent (sliding) or permanent (fixed) [1–5]. Loop stomas are more prone to prolapse compared to end stomas, and ileostomies are at higher risk compared to colostomies [6]. Risk factors for stoma prolapse include conditions associated with increased intra-abdominal pressure, such as obesity and chronic obstructive pulmonary disease, redundancy of bowel, lax connective tissue and technical errors, such as foregoing preoperative stoma site marking and creating an overly large stoma fascial aperture [3]. The majority of prolapses are sliding and uncomplicated and are managed conservatively [5]. However, more extensive prolapse that significantly impact the quality of life or lead to complications, such as ulceration, bleeding, ischemia, incarceration and obstruction, may require surgical correction.

Closure of the stoma and restoration of gastrointestinal continuity is the most obvious solution but is not possible for many patients. Numerous surgical techniques have been described for stomal prolapse, including parastomal procedures, such as resection of the prolapsed bowel and conversion of a loop to an end stoma and transabdominal procedures, such as enteropexy and stoma resiting. Unfortunately, recurrence rates remain high regardless of the surgical technique [7].

The modified laparoscopic Sugarbaker procedure is well established for repair of parastomal hernia [8]. We describe here a novel application of this operation for the management of a recurrent prolapse of an end ileostomy.

## MATERIALS AND METHODS

**Figure 1:**
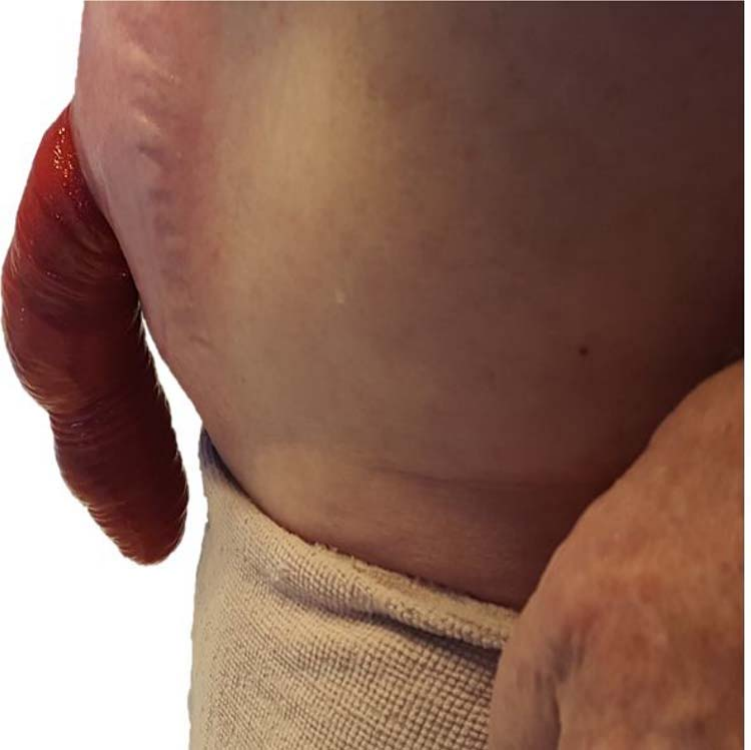
Ileostomy prolapse.

A 57-year-old male was referred with recurrent ileostomy prolapse. His index operation was a proctocolectomy for ulcerative colitis which was performed ~30 years prior. He presented with a permanently prolapsed stoma that significantly impacted his quality of life, including his ability to work and his relationship with his spouse. He had no significant medical co-morbidities and no history of smoking. Apart from the proctocolectomy, his surgical history was significant for four surgeries for stomal prolapse; including three local revisions and resiting of the stoma to the left flank. On each occasion, he noted recurrence of the prolapse within the first 3 months post-operatively. On clinical examination, he was of average build with a body mass index of 25.1, and he had no marfanoid features or other stigmata of connective tissue disease. Abdominal exam revealed a fixed stomal prolapse of ~30 cm (Fig. 1) and a well-healed midline laparotomy scar.

After extensive discussion, the patient consented to a modified laparoscopic Sugarbaker procedure. He was informed that this was a novel application of a procedure designed for repair of parastomal hernia.

In anticipation of significant intra-abdominal adhesions given his prior surgeries, we performed a preoperative ileoscopy and tattooed the stomal limb to aid with laparoscopic identification.

**Figure 2:**
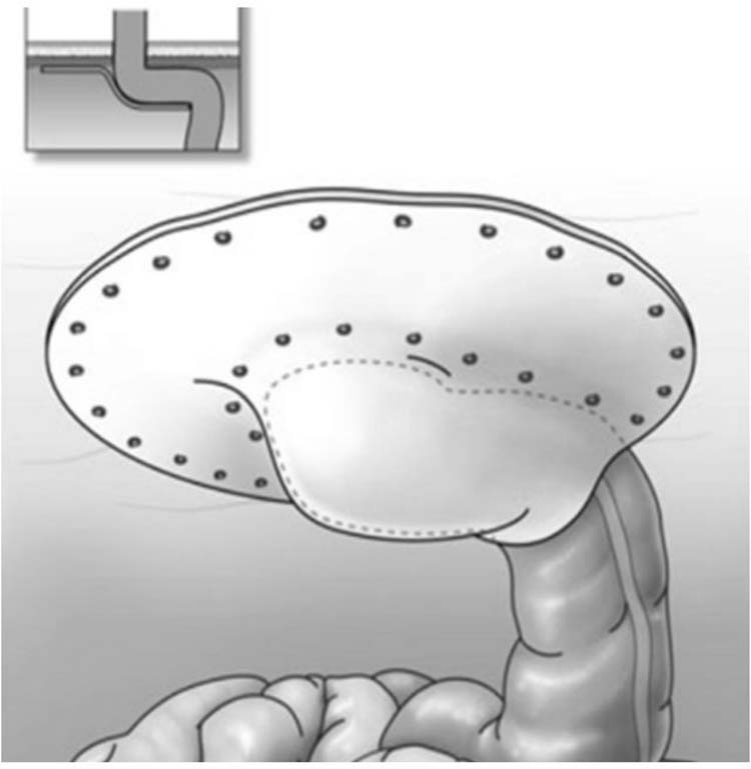
Modified laparoscopic Sugarbaker procedure.

At surgery, following complete lysis of adhesions, we were able to identify the stomal limb by submucosal tattoo. We pexied the stomal limb to the anterior abdominal wall, from the stoma facial opening across the midline to the right flank, with four interrupted absorbable sutures using the intracorporeal technique. When performing the Sugarbaker procedure, we routinely pexied the stomal limb to the abdominal wall, as we found this to facilitate optimal positioning and securing of the mesh. A 26 × 34-cm expanded polytetrafluoroethylene mesh (Dulex™, C.R. Bard, Murray Hill, NJ) was trimmed to size and positioned over the pexied bowel in Sugarbaker fashion (Fig. 2) [9]. We then secured the mesh to the anterior abdominal wall using titanium tacks (ProTack™, Medtronic, Dublin, Ireland).

The patient was discharged on post-operative day 3, and at the time, he had minimal pain, his stoma was functioning and he tolerated a regular diet.

## RESULTS

**Figure 3:**
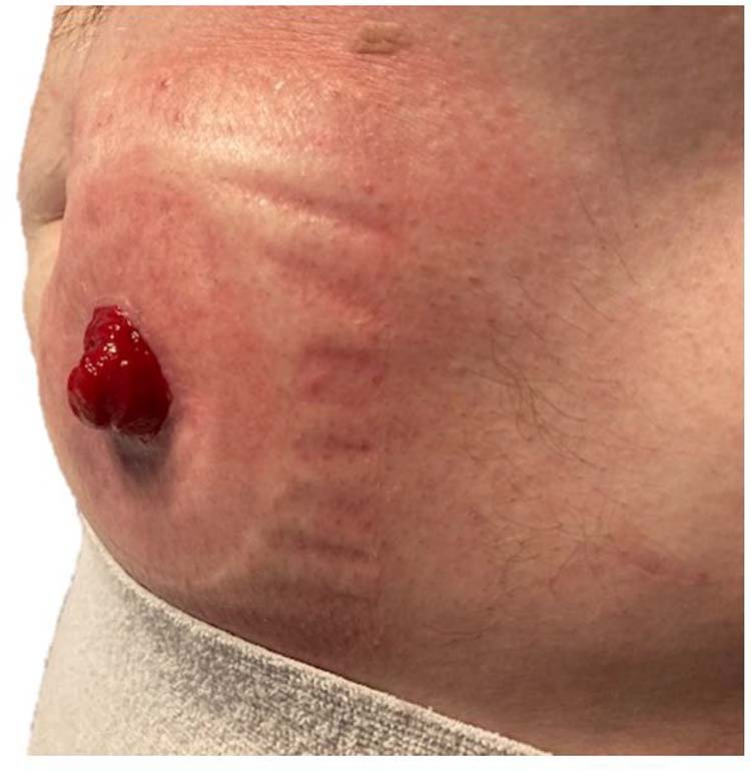
Ileostomy 1-year post-modified laparoscopic Sugarbaker repair of prolapse.

On 1-year follow-up, the stoma remained healthy with no recurrence of the prolapse (Fig. 3). The patient was able to resume his work in demolition and had significant improvement in his intimate relationship.

## DISCUSSION

Stomal prolapse is a common complication of intestinal stomas which can cause significant morbidity and complications that may necessitate surgical repair. However, current techniques are associated with high recurrence rates [10]. We have considerable experience with the modified laparoscopic Sugarbaker procedure in repair of parastomal hernias, and we hypothesized that the lateralization and splinting of the bowel inherent to this operation could resolve the recurrent prolapse of an end ileostomy.

## CONCLUSION

This case demonstrates the novel application of the modified laparoscopic Sugarbaker procedure for the repair of a recurrent stomal prolapse and provides an alternative option for the surgeon managing this challenging clinical problem. Further studies are needed to determine longer-term outcomes compared to established techniques.

## CONFLICT OF INTEREST STATEMENT

None of the authors have a conflict of interest to report.

## FUNDING

No funding was received for this study.
